# Editorial: Catalysis and sensing for our environment

**DOI:** 10.3389/fchem.2025.1660145

**Published:** 2025-07-23

**Authors:** Tony D. James, William D. G. Brittain, Daniel T. Payne, Alexander J. Cresswell, Tsuyoshi Minami, Robert B. P. Elmes

**Affiliations:** ^1^Department of Chemistry, University of Bath, Bath, United Kingdom; ^2^Chemistry Department, Durham University, Durham, United Kingdom; ^3^School of Life, Health and Chemical Sciences, The Open University, Milton Keynes, United Kingdom; ^4^Institute of Industrial Science, The University of Tokyo, Tokyo, Japan; ^5^Department of Chemistry, Maynooth University, National University of Ireland, Maynooth, Ireland

**Keywords:** catalysis, sensing, environment, molecular recognition, transformations

This Research Topic has been assembled in collaboration with Frontiers in Chemistry to honour the memory of our friend and colleague, Professor John S. Fossey (University of Birmingham). It coincides with the 10th Catalysis and Sensing for our Environment (CASE) symposium, held in Japan from 5 to 12 April 2024—an event that John had long envisioned, in a country that held deep personal and professional significance for him.

John was one of the driving forces behind the CASE Network, co-founding it in 2008 alongside Professors Tony D. James and Steven D. Bull. Since its inception, the CASE Network has brought together an international community of researchers in catalysis and sensing, fostering collaboration and innovation across continents. The symposium has been hosted in the United Kingdom, Ireland, the US, China, and in 2024, for the first time, across multiple institutions in Japan:

5April: University of Kitakyushu–Local Organizer: Kazuo Sakurai.

8April: Osaka University–Local Organizer: Kazuya Kikuchi.

10April: Tokyo Metropolitan University–Local Organizers: Yuji Kubo & Tsuyoshi Minami.

11April: International Center for Materials Nanoarchitectonics (MANA) – Local Organizer: Jonathan Hill.

12April: University of Tokyo–Local Organizer: Shū Kobayashi.

These meetings reflect the global and interdisciplinary spirit of CASE, a network born of John’s commitment to meaningful scientific exchange.

The themes of catalysis and sensing—two fields in which John made enduring contributions—are deeply interconnected. Catalysts enable selective molecular transformations that are foundational in developing pharmaceuticals and sustainable technologies. Sensors, by contrast, translate molecular recognition events into measurable signals, playing crucial roles in biological, clinical, and environmental monitoring. Though their goals differ, both rely on precise molecular interactions, and many advances in one field inform progress in the other.

The goal of this Research Topic is to present recent developments emerging from the intersection of catalysis and sensing. This Research Topic features two reviews and five research articles. We hope this compilation strengthens interdisciplinary ties and continues to build upon the collaborative foundations that John so passionately helped establish.

Professor John S. Fossey earned his chemistry degree in 2000 from Cardiff University, and his PhD from Queen Mary University of London in 2004 under the supervision of Professor Christopher J. Richards. He then moved to Japan as a JSPS postdoctoral fellow with Professor Shū Kobayashi at the University of Tokyo. During his time in Japan, he met his future wife, Rumi Fossey (née Matsumoto), and together they raised three children: Karen, Toby, and Edwin.

After returning to the United Kingdom in 2005, John held a position at the University of Bath before joining the University of Birmingham. He remained deeply connected to Japan throughout his life, both personally and professionally, and worked tirelessly to bridge scientific communities through the CASE network. His vision for a roving, international symposium was fully realized in this 10th meeting—culminating in a heartfelt and fitting tribute.

Significantly on 12 April 2024, at the University of Tokyo, Professor Yun-Bao Jiang one of John’s close friends was awarded the inaugural John S. Fossey CASE Award, presented by Professor Shū Kobayashi. This award recognizes outstanding contributions to the CASE community and commemorates John’s legacy of excellence, generosity, and global scientific partnership.

We dedicate this Research Topic to John’s memory and to the enduring impact of his work. Through these papers, we celebrate not only scientific progress but the collaborative spirit he championed.

## Overview of papers contained in this Research Topic

### A systematic review of sensors to combat crime and routes to further sensor development

This systematic review examines sensor technologies used to combat crime, highlighting current challenges and opportunities for future development. Out of 1,482 publications reviewed, 791 focused on low-cost sensing devices published since 2020. Eleven key analyte categories were identified, including illicit drugs, body fluids, explosives, and fingerprints. Low-cost technologies were classified as electrochemical, colorimetric, immunoassay, luminescence, and SERS-based (Cozens et al.).

The review found that current forensic sensors often suffer from high costs, limited specificity, and complex procedures, making them reliant on trained professionals. Emerging research emphasizes printed electrodes and dual detection systems to improve sensitivity and accuracy. Body fluid analysis and illicit drug detection were particularly prominent areas, with ongoing development aimed at improving specificity, reducing false positives, and lowering costs.

To advance the field, the review recommends focusing on eco-friendly materials, reducing matrix interference, refining dual detection techniques, and strengthening partnerships between researchers and law enforcement for real-world application.

### Metal ion-manipulated afterglow on rhodamine 6G derivative-doped room-temperature phosphorescent PVA films

This study presents the design of room-temperature phosphorescent (RTP) PVA films that exhibit tunable afterglow in response to metal ions. Two RTP-active films, TDB@PVA and ATB@PVA, were created via boronate esterification and showed turquoise and green afterglow, respectively. Doping the films with a spirolactam-based rhodamine 6G derivative enabled metal-ion-triggered multicolor emission through a triplet-to-singlet Förster-type energy transfer mechanism. Upon coordination with metal ions, the dye switches from a non-fluorescent to a fluorescent state, allowing reversible write/erase optical patterns. This work highlights potential applications in data encryption, anti-counterfeiting, and smart luminescent materials (Kubo et al.).

### Highly thermostable RhB@Zr-Eddc for the selective sensing of nitrofurazone and efficient white light emitting diode

This study reports the synthesis of a highly thermostable composite, RhB@Zr-Eddc, created by encapsulating Rhodamine B within the nanocages of Zr-Eddc via a one-pot hydrothermal method. Characterized by XRD, SEM, and TGA, the material demonstrates thermal stability up to 550°C and bright red emission at 605 nm. RhB@Zr-Eddc selectively detects the antibiotic nitrofurazone in water among eleven tested antibiotics. Additionally, when combined with a green phosphor and applied to a 455 nm blue LED chip, the composite produces an efficient white light-emitting diode (WLED) with a CCT of 4710 K, luminous efficiency of 43.17 Lm/W, and a high CRI of 89.2 (Shen et al.).

### Utilising a 1,8-naphthalimide probe for the ratiometric fluorescent visualisation of caspase-3

This study presents a novel ratiometric fluorescent probe, Ac-DEVD-PABC-Naph, designed to detect caspase-3 activity, a key marker of apoptosis. The probe features a 1,8-naphthalimide fluorophore linked to a peptide substrate via a self-immolative PABA linker. Upon cleavage by caspase-3, the probe releases the free fluorophore, triggering a ratiometric fluorescence shift. Spectroscopic and kinetic analyses confirmed its high sensitivity, selectivity for caspase-3, and effective detection limits. With its ratiometric readout and strong specificity, the probe shows promise for visualising caspase-3 activity in biological systems, offering potential for apoptosis research and therapeutic development (Elmes and Wynne).

### Development of elliptic core-shell nanoparticles with fluorinated surfactants for ^19^F MRI

This study reports the development of elliptic core-shell silica nanoparticles encapsulating perfluoro-15-crown-5 ether (PFCE) for use as sensitive probes in ^19^F MRI. Synthesized using fluorinated surfactants C10-TAC and C8-TAC, the resulting PFCE@SiO_2_ nanoparticles exhibit an elliptic morphology and strong ^19^F NMR signals. Their distinct shape and robust fluorine signal make them a promising alternative to traditional spherical nanoparticles for advanced fluorine-based bioimaging (Kikuchi et al.).

### Research progress in the detection of trace heavy metal ions in food samples

This mini-review highlights recent advances in detecting trace heavy metal ions in food, a critical Research Topic due to their high toxicity and threat to public health. It focuses on analytical methods using nanomaterials, categorized into electrochemical, colorimetric, and fluorescent techniques. The review outlines the sensing mechanisms and features of each method, supported by practical examples, and discusses their strengths, challenges, and future prospects. By summarizing current progress, the review aims to support the development of more effective and sensitive detection technologies for ensuring food safety (Jin et al.).

